# Influence of different urine preservation methods and addition of preservatives on quantitative detection of 24-h urinary protein

**DOI:** 10.3389/fmed.2025.1661339

**Published:** 2025-11-07

**Authors:** Xinxin Bao, Xiaoye Sun, Haiying Geng, Xiaohua Yuan

**Affiliations:** 1 Department of Clinical Laboratory, Affiliated Maternity and Child Health Care Hospital of Nantong University, Nantong, China; 2 Department of Blood Transfusion, Affiliated Hospital of Nantong University, Nantong, China; 3 Department of Blood Transfusion, Affiliated Maternity and Child Health Care Hospital of Nantong University, Nantong, China

**Keywords:** 24-h urinary protein, preservation, xylene, quantitative detection, sample stability

## Abstract

**Objective:**

This study aimed to elucidate the effect of adding xylene as a preservative on 24-h urine protein quantification under different storage temperatures.

**Methods:**

From January 2020 to August 2020, our hospital selected a total of 80 samples with positive results of urine protein. Under different storage temperature conditions, urine samples containing or not containing xylene were collected simultaneously. Then, one-way analysis of variance was used to study the effects of preservatives and temperature on the 24-h urine protein quantification test. Receiver operating characteristic (ROC) analysis was used to examine the effect of adding preservatives on the accuracy of the 24-h urinary protein concentration determination.

**Results:**

Based on the results of the control group, there was no statistically significant difference in the 24-h urine protein concentration between the preservative group and the group without preservatives at 37, 24–26 or 4 °C (*F* = 0.006, *P* = 0.993; *F* = 0.013, *P* = 0.987; *F* = 0.022, *P* = 0.977). The results of the ROC analysis indicated excellent diagnostic accuracy for proteinuria detection across all storage conditions (AUC: 0.992–0.994). The accuracy of urine samples stored without preservatives was comparable to, and in some cases (e.g., at 4 °C) exhibited perfect specificity (100%) alongside high sensitivity (97.4%), matching the direct detection in the control group.

**Conclusion:**

Storing 24-h urine protein specimens at room temperature without using preservatives is a safe, simple, and feasible method. This method is suitable for wide application in clinical practice.

## 1 Introduction

The 24-h urine protein quantification is one of the core indicators for evaluating kidney function, diagnosing kidney diseases, and monitoring treatment effects (1). By collecting all the urine within 24 h and accurately measuring the total amount of protein in it, it can more comprehensively reflect the filtration and reabsorption functions of the kidneys for proteins, avoiding errors caused by urine concentration or dilution in a single urine test (2). Proteinuria refers to a pathological phenomenon where the protein content in urine is abnormally elevated. In the urine of healthy individuals, only trace amounts of protein are present (the 24-h excretion is < 150 mg) (3). When the protein content in the urine exceeds this range, or when the random urine protein/creatinine ratio is >200 mg/g, it is called proteinuria. Proteinuria is closely related to kidney diseases (4). It is not only a sign of kidney damage but also an important factor that accelerates kidney damage (5). Therefore, 24-h urine protein testing is the “gold standard” for evaluating renal function. The test results are helpful for disease diagnosis, predicting disease progression, and providing scientific basis for formulating treatment plans (6). Due to the unstable excretion volume of urine protein and its diverse physical and chemical properties, it is crucial to select the correct specimen collection and preservation methods.

The process of collecting and preserving urine samples is influenced by various factors, such as temperature, preservatives, preservation methods, preservation time, environmental conditions, drug metabolites, and pigments in the urine (7). Therefore, understanding the factors that affect the 24-h urine protein test, choosing the method that has the least impact on the test results, and minimizing or avoiding the factors that may have a significant influence on the test results are of crucial importance for the diagnosis and treatment of the disease.

The storage methods for urine samples include refrigeration and chemical preservation treatment (8, 9). Chemical preservatives are used to preserve urine by inhibiting bacterial growth (10). Common preservatives include toluene, xylene, boric acid, hydrochloric acid, and thymol (11). Toluene and xylene, these two substances, are the most commonly used preservatives in qualitative or quantitative analysis of chemical components (such as proteins in urine). They can also play a role in preserving urine samples collected at a certain time or those that cannot be tested promptly (12, 13). Although preservatives such as boric acid and mercurochrome are considered safer, their limitations in urine protein testing are significant: boric acid can cause long-term protein hydrolysis, mercurochrome interferes with the heating method for protein detection, and sodium fluoride is only suitable for stabilizing urine sugar (14, 15). In contrast, xylene can effectively inhibit the reproduction of aerobic bacteria by forming a physical barrier, and at the recommended dose, it does not interfere with the results of the diazo method (16). Nevertheless, preservatives like xylene have some drawbacks, such as being carcinogenic, difficult to precisely control the dosage, and having an impact on sample absorption during testing (17). Therefore, despite the widespread use of xylene, concerns regarding its carcinogenicity, difficulty in precise dosing, potential interference with assays, and safety for staff/patients create a need to evaluate its necessity.

This study aimed to elucidate the effect of the presence or absence of xylene on the 24-h urine protein quantification test under common storage temperatures (4 °C, room temperature, 24–26, and 37 °C). The methods included directly preserving the urine samples, detecting the urine protein concentration after collection, and testing the urine protein concentration under room temperature, temperature-controlled cabinets, or refrigerated conditions. This may offer a clinically relevant foundation for finding more suitable clinical urine preservation methods.

## 2 Materials and methods

### 2.1 General data

From January 2020 to August 2020, our hospital selected a total of 80 samples with positive results of urine protein. This study complied with the Declaration of Helsinki, and all participants signed the informed consent form. This study was approved by the ethics committee of Affiliated Maternity and Child Health Care Hospital of Nantong University, and the approval number was Y2022027.

### 2.2 Specimen collection

The 24-h urine samples were collected from 80 different samples to create a mixed urine sample. The specific procedure was as follows: each patient was informed in advance about the requirements and precautions for collecting urine, ensuring that they understood and cooperated. During the collection process, the patient used clean and dedicated urine collection containers. After collecting the urine samples from these 80 samples, they were thoroughly mixed. To ensure a thorough mixture, all the urine was first poured into a large, clean container, and then a sterile stirring rod was used to stir slowly for 10 min. This process allowed the urine samples from different patients to fully blend and form a combined urine sample.

The dry chemical test results indicated that the level of urine protein was between 1^+^ and 3^+^. Urine protein 1^+^ indicated that 1 L of urine contained 0.2–1 g of protein, which was expressed as 0.2–1 g/L; urine protein 2^+^ indicated that 1 L of urine contains 1–2 g of protein, which was expressed as 1–2 g/L; urine protein 3^+^ indicated that 1 L of urine contained 2–4 g/L of protein, which was expressed as 2–4 g/L. After mixing, the urine specimens were divided into six urine sampling tubes, each containing 10 ml of mixed urine. Under each storage condition (24–26 °C: room temperature, 37 °C: temperature box, and 4 °C: refrigeration), the specimens were placed into two tubes simultaneously. One tube was sealed (without preservatives), and the other was covered with xylene (10 ml of urine plus ~100 μl of xylene; preservative group). After the urine specimen was left for 24 h, all the samples were subjected to centrifugation at a speed of 3,000 *r*/min for 10 min at room temperature, and then the concentration of urine protein was detected. A portion of the mixed urine was collected and immediately centrifuged to determine the urine protein concentration (control group). The urine protein concentration of 0–0.14 g/L was considered within the normal range. When the quantification of urine protein exceeded 0.15 g/L, it was considered that the urine protein content was increased.

### 2.3 Instruments and reagents

For 37 °C: carbon dioxide incubator (Thermo; model No. 3111); 4 °C: blood refrigerator (Haier) was used; room temperature: 24–26 °C was maintained. A Beckman AU640 Fully Automatic Biochemical Analyzer (Beckman Coulter, USA), a Cerebrospinal Fluid and Urinary Protein Assay Kit (Beijing Leadman Biochemistry Co., Ltd., Lot number: 19071601, Expiry date: 2020-12-11), and xylene (Changshu Hongsheng Fine Chemical Co., Ltd., Lot number: CY20200125, Expiry date: 2021-01-25) were used.

### 2.4 Testing methods

The instrument calibration was completed, and the internal quality control met the requirements. All urine specimens were centrifuged, and the supernatant was collected. Urine protein was detected using the pyrogallol red-molybdate method. This method is based on the formation of a red complex (with a maximum absorption peak of 467 nm) between phenolphthalein red (PR) and molybdate under acidic conditions. When this complex combines with proteins in urine, its molecular structure changes, causing the maximum absorption peak to shift to 594–600 nm. At this point, the absorbance at the 600 nm wavelength is linearly related to the protein concentration. By performing colorimetric analysis against a standard protein solution, the protein content in urine can be quantitatively determined.

### 2.5 Protein calibration standards

Firstly, we used standard calibration products that were compatible with the instrument and had undergone strict quality inspection and certification. The protein concentration values of these calibration products were accurate and reliable. The instrument automatically recorded the detection signals and compared them with the standard values for analysis. Through built-in algorithms, the detection parameters of the instrument were adjusted to ensure that the error between the instrument's detection value and the standard value remains within a very small range. After multiple calibrations and verifications, when the relative error of the instrument's detection result compared to the standard value was ≤ ±15% each time, we considered the instrument calibration to be qualified and could proceed with subsequent sample testing. Additionally, during the experiment, we also regularly used standard calibration products to recheck the instrument to ensure that it maintained a good calibration status throughout the experiment, thereby ensuring the accuracy and reliability of the urine protein concentration detection results.

### 2.6 Quality control measures

To ensure the accuracy and reliability of the experimental results, we implemented strict internal quality control measures. The preparation process of the quality control samples strictly followed the relevant standard operating procedures to ensure the uniformity and stability of the concentration. During each batch of sample testing, we performed centrifugation, collected the supernatant, and conducted protein concentration tests together with the quality control samples. If the test results of the quality control samples exceeded the pre-set quality control standard range, it could be concluded that there was an abnormal situation in this batch of experiments. We would immediately stop the experiment, investigate possible causes, such as instrument malfunctions, reagent issues, or operational errors, and re-perform the experiment after solving the problems until the test results of the quality control samples met the requirements, and then continue to test the actual urine samples. Through this strict quality control measure, we can effectively monitor the stability and accuracy of the experimental process and ensure the reliability of the protein concentration test results for each batch of urine samples.

### 2.7 Statistical analysis

Statistical analyses were performed using SPSS 27.0 software. The measurement data were represented as (*x* ± *s*) and then analyzed using a one-way analysis of variance for comparisons. The count data were represented as (*n*, %) and then analyzed using the chi-square test for comparisons. In order to deeply explore the impact of different temperature conditions on the accuracy of 24-h proteinuria testing, we plotted and analyzed the receiver operating characteristic (ROC) curve, clearly identifying the efficacy of the 24-h proteinuria testing method in differentiating between proteinuria-positive and proteinuria-negative patients under various temperature settings. The pyrogallol red-molybdate method was used to as the gold standard. In this study, a control group was set up for immediate testing after sample collection. After completing the 24-h urine collection, an appropriate amount of urine sample was taken immediately from it. According to the standardized testing procedure, using the same testing methods and reagents as those in the subsequent different temperature treatment groups, the proteinuria content was determined within the specified testing time. This ensures that the testing of the control group samples is not affected by temperature changes, providing a reliable benchmark for comparing the accuracy of test results under different temperature treatments in the subsequent stages. When drawing the ROC curve, the commonly used clinical proteinuria diagnostic threshold of 0.15g/24h was used as the initial threshold for the preliminary analysis. Statistical significance was set at *P* < 0.05.

## 3 Results

### 3.1 Absence of xylene exerts no impact on 24-h urinary protein concentration at 37 °C

**Figure 1:**
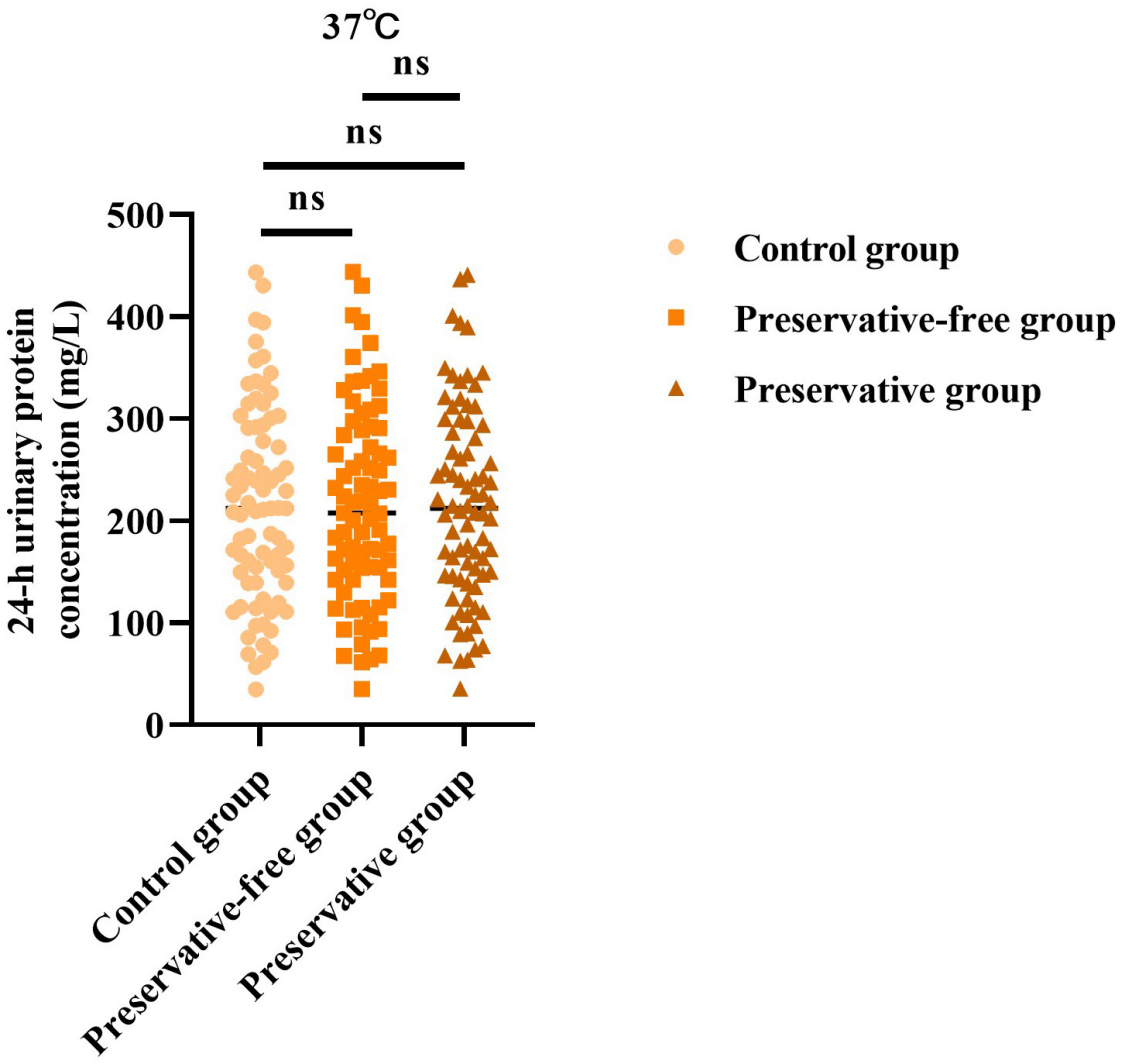
24-h urinary protein concentration in each group at 37 °C. ns = no significance.

Based on the results of the control group, there was no statistically significant difference in the 24-h urine protein concentration between the group using preservatives and the group not using preservatives at 37 °C (*F* = 0.006, *P* = 0.993; Figure 1). This indicated that, even without the use of preservatives, when urine samples were placed in an environment at 37 °C, the quantitative determination results of urine protein concentration were not affected.

### 3.2 Absence of xylene exerts no impact on 24-h urinary protein concentration at 24–26 °C

**Figure 2:**
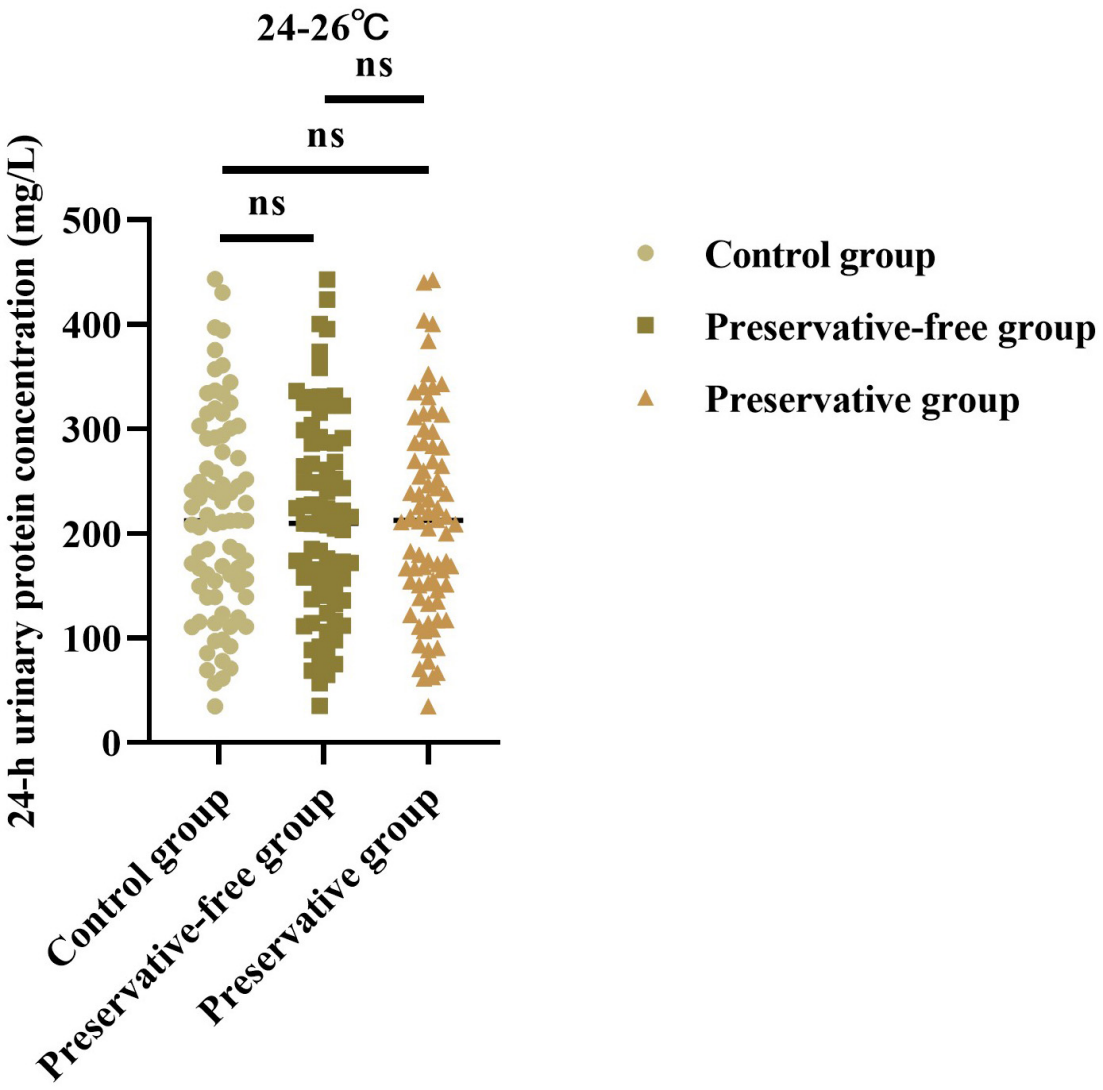
24-h urinary protein concentration in each group at 4 °C. ns = no significance.

Based on the results of the control group, there was no statistically significant difference in the 24-h urine protein concentration between the group using preservatives and the group not using preservatives at 24–26 °C (*F* = 0.013, *P* = 0.987; Figure 2). This indicated that, even without the use of preservatives, when urine samples were placed in an environment at room temperature, the quantitative determination results of urine protein concentration were not affected.

### 3.3 Absence of xylene exerts no impact on 24-h urinary protein concentration at 4 °C

**Figure 3:**
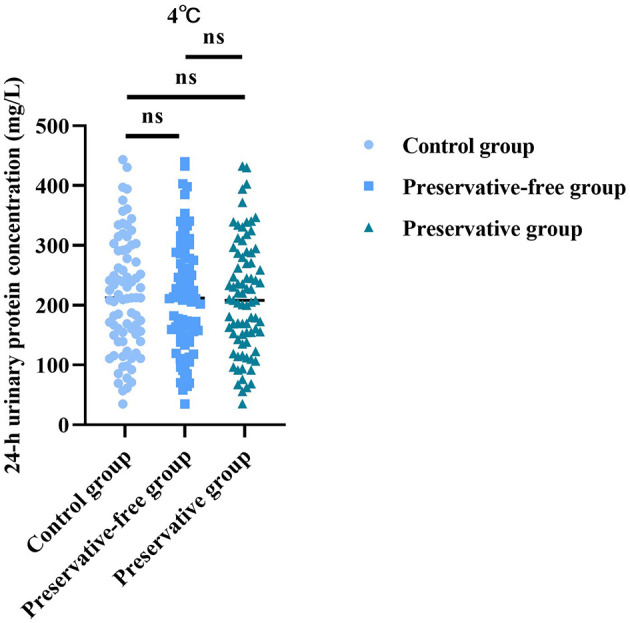
ROC curve analysis of the accuracy of detection of 24-h proteinuria at different temperatures.

Based on the results of the control group, there was no statistically significant difference in the 24-h urine protein concentration between the group using preservatives and the group not using preservatives at 4 °C (*F* = 0.022, *P* = 0.977; Figure 3). This indicated that, even without the use of preservatives, when urine samples were placed in a refrigerated condition, the quantitative determination results of urine protein concentration were not affected.

### 3.4 Absence of xylene exerts no impact on the detection of 24-h proteinuria at different temperatures

**Figure 4:**
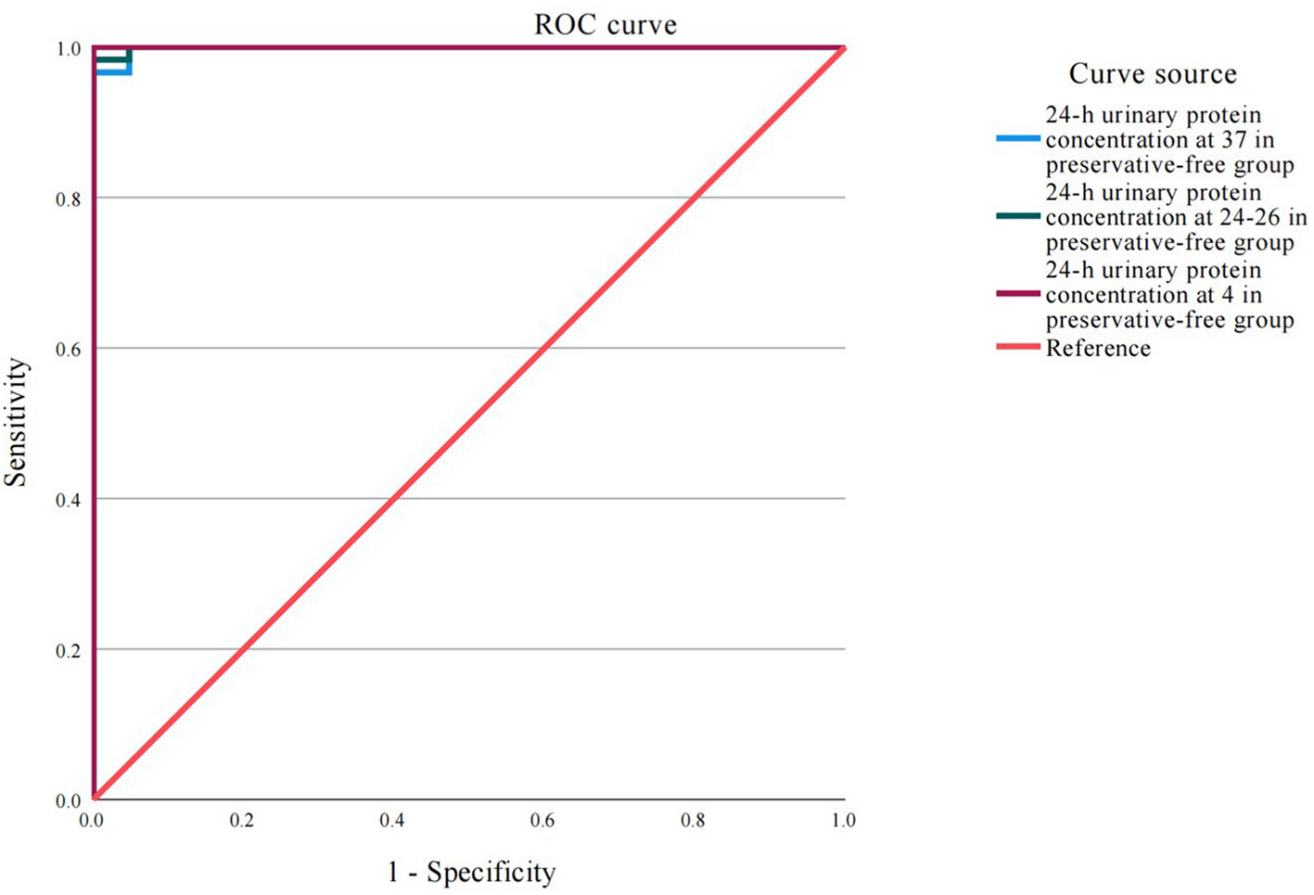
24-h urinary protein concentration in each group at 24–26 °C. ns = no significance.

The ROC analysis results demonstrated that under different storage temperatures (37, 24–26, and 4 °C), the accuracy of detecting proteinuria in 24-h urine samples, whether with or without xylene preservative, was excellent and comparable to that of direct proteinuria detection in the control group (Figure 4 and Table 1). The area under the curve (AUC) values for all six experimental conditions ranged from 0.992 to 0.994 (all *P* < 0.001). Notably, the sensitivity remained consistently high at 97.4% across all groups, while specificity reached 100% in the 24–26 °C with xylene, 4 °C with xylene, and 4 °C without preservative groups.

**Table 1:** ROC curve analysis results of the accuracy of detection of 24-h proteinuria at different temperatures.

**Storage condition**	**AUC (95% CI)**	**Optimal threshold (mg/L)**	**Sensitivity**	**Specificity**	***P*-value**
37 °C with xylene	0.992 (0.975–1.000)	152.6	0.974	0.971	< 0.001
37 °C without preservative	0.993 (0.978–1.000)	154.2	0.974	0.971	< 0.001
24–26 °C with xylene	0.993 (0.979–1.000)	152.3	0.974	1.000	< 0.001
24–26 °C without preservative	0.992 (0.976–1.000)	151.9	0.974	0.971	< 0.001
4 °C with xylene	0.994 (0.981–1.000)	151.1	0.974	1.000	< 0.001
4 °C without preservative	0.993 (0.978–1.000)	150.5	0.974	1.000	< 0.001

## 4 Discussion

The 24-h urinary protein is an important indicator for evaluating changes in kidney function. It holds extremely significant clinical importance for the diagnosis, treatment and prognosis of kidney diseases (18). Due to the long preservation time of 24-h urine specimens, changes in environmental temperature during the preservation process often have an impact on the bacterial growth and metabolism in the urine, thereby affecting the quality of the urine samples (19). Therefore, correctly storing 24-h urine specimens is a prerequisite for obtaining accurate results.

In clinical practice, xylene or toluene is commonly used as a preservative to store 24-h urine protein test samples. The main function of these preservatives is to form a layer on the surface of the liquid to isolate the sample from the air, thereby achieving the purpose of preservation (20). Studies has shown that lower concentrations of toluene can be used for preservative treatment in 24-h urine (21). However, the use of xylene (an organic substance that is insoluble in water) may result in a lower detection value for urine protein, because the amount of sample added during sampling and testing with an automatic biochemical analyzer has been reduced (22). Furthermore, xylene is a highly pungent carcinogenic substance that poses potential health risks to humans, and its usage is difficult to precisely control (23). Thus, finding a simple and safe method for urine preservation is of great significance for ensuring the accuracy of test results and reducing the harm that preservatives may cause to the human body.

This study explored the impact of preservatives on 24-h urinary protein concentration from the perspective of environmental temperature. The results showed that, regardless of whether xylene was added to the urine as a preservative for storage, no statistically significant difference was observed between the measured values of urine protein concentration after 24 h of storage and the immediate test results under different environmental temperature conditions. This study also indicated that, under four different temperature conditions (4, 24–26, and 37 °C), the absence of preservatives in urine samples had almost no impact on the measurement results of urine protein concentration. These findings were highly consistent with the relevant content of WHO international clinical guidelines (24). The WHO guidelines emphasize the importance of ensuring the accuracy of test results during the collection and storage of urine samples. Although this guideline does not provide detailed explanations regarding the specific temperature conditions and preservative usage involved in this study, its core principle is to ensure the stability of urine samples from collection to testing, in order to obtain reliable measurements of urine protein concentration. The results of this study indicate that in various environmental temperatures, neither adding preservatives nor using xylene did significantly affect the measurement of urine protein concentration. This, in turn, supports the overall requirements of the WHO guidelines for sample stability. That is, regardless of the preservation method (adding or not adding preservatives), as long as the sample can be maintained stable within a reasonable temperature range, accurate test results can be obtained, which aligns with the goal pursued by the WHO guidelines. Consistent with our findings, a previous report indicated that urine stored at low temperature and urine stored at room temperature could inhibit and facilitate bacterial growth and reproduction, respectively, but neither of them affected the accuracy of urine protein concentration measurements (25). Therefore, the author suggested that the samples used for 24-h urine protein measurement were stored at room temperature (26).

The results of our comprehensive ROC analysis provide robust statistical evidence supporting the main findings. The exceptionally high AUC values (all >0.99), coupled with consistently high sensitivity (97.4%) and specificity (reaching 100% in three out of six conditions), indicate that the urine protein quantification is remarkably stable under various storage scenarios. Crucially, the performance metrics for samples stored without any preservative were virtually identical to those preserved with xylene. This finding is particularly significant as it demonstrates that the simple omission of a preservative does not compromise the analytical integrity of the 24-h urine protein test, even when samples are stored at room temperature for 24 h. Therefore, this study reaches the following conclusion: the 24-h urine protein test samples can be stored at room temperature without the need for the addition of preservatives. If due to instrument failure or other reasons, timely testing cannot be conducted, it is recommended to store them in a refrigerated environment to extend their shelf life and improve the accuracy of the 24-h urine protein test results. Consistent with our findings, studies have shown that even when sodium chloride preservatives were added to the urine, the measurement results of urinary protein and microalbumin in 24-h urine did not show significant improvement. Moreover, even without adding preservatives to the urine, the test results still had a good correlation with the standard measurement results (27).

Our study has some limitations. First, this study did not include samples with microalbuminuria, so the conclusions are not applicable to scenarios such as early screening for diabetic nephropathy where there is low levels of proteinuria. Future research needs to combine high-sensitivity detection methods to further evaluate the interference effect of xylene. Second, this study did not conduct dose-effect experiments to test the impact of different amounts of xylene on the stability of urine protein, the centrifugation effect, and the interference of detection. Future research should further clarify the dose-interference relationship of xylene to provide a more precise basis for the selection of clinical preservatives. Third, this study did not monitor the storage temperature in real time, which may have underestimated the impact of extreme temperature fluctuations (such as power outages or equipment failures) on the results. Future research should combine intelligent temperature recording technology to further verify the reliability of the results under non-ideal conditions. Fourth, this study only evaluated the sample preservation stability within 24 h. However, in actual clinical operations, due to various unforeseen factors such as equipment malfunctions and sample transportation delays, the testing time of the samples may be delayed, and the preservation period may be extended to 48–72 h or even longer. Nevertheless, this study did not conduct in-depth exploration of the stability of samples beyond 24 h. This may lead to the situation that in clinical practical applications, for samples that need long-term preservation, it is impossible to accurately judge the feasibility and reliability of preserving them without preservatives at room temperature, thereby limiting the wide applicability of the conclusions of this study. Future research should further extend the observation range of sample preservation time, comprehensively evaluate the stability of samples under different preservation durations, and provide more comprehensive sample preservation guidance for clinical practice. Fifth, a total of 80 samples were included in this study, and from the perspective of sample size, it was relatively limited. This study may not have adequately considered the influence of the internal variability and individual differences within the sample on the research results. A smaller sample size and inappropriate selection of statistical units may lead to deviations in the research results, reducing the reliability and persuasiveness of the research conclusions. Subsequent studies should increase the sample size, while reasonably determining the statistical units, fully considering various factors that may affect the research results, in order to improve the quality and accuracy of the research. Sixth, during the storage of urine samples, bacterial growth is one of the main reasons for the addition of preservatives. Preservatives can inhibit bacterial reproduction and prevent bacteria from decomposing the proteins and other components in the urine, thereby ensuring the accuracy of urine protein test results. However, this study lacks microbial culture data and failed to monitor and analyze the bacterial growth situation in the samples. This makes it impossible for us to know exactly the bacterial growth status of the samples during storage and its impact on the stability of urine protein. The lack of this key data weakens the credibility of the statement “no need to use preservatives” in this study. Future research should add bacterial count detection items and comprehensively evaluate the bacterial growth situation in the samples under different storage conditions through microbial culture and other methods, providing a more solid basis for the selection of sample storage methods. Seventh, this study has certain flaws in statistical analysis, lacking true patient-level repetitive analysis. Patient-level repetitive analysis can better reflect the stability of samples of individual patients at different time points or under different conditions, and is helpful for more accurately evaluating the effectiveness and reliability of preservation methods. Due to the lack of this analysis, we cannot determine whether the stability and consistency of the research results are significantly affected by individual differences. Future research should strengthen patient-level repetitive analysis, adopt more scientific and reasonable statistical methods, fully consider the influence of individual differences on the research results, in order to improve the scientificity and practicability of the research. Eighth, bacterial count and pH are important factors affecting the quality of urine samples. They change over time and thereby influence the stability of urine protein. However, this study did not assess the changes of bacterial count and pH over time, and thus could not confirm the changes of these indicators under different preservation conditions (with or without preservatives) and their effects on the preservation of urine protein. Future research should increase the monitoring and analysis of the changes of bacterial count and pH over time, and comprehensively understand the relationship between these indicators and the stability of urine protein, in order to provide more comprehensive information for optimizing sample preservation methods.

In conclusion, our data demonstrate that omitting xylene as a preservative does not affect the quantitative accuracy of the 24-h urinary protein test, as validated by superior ROC performance metrics (AUC >0.99, Sensitivity 97.4%) across a range of storage temperatures. Directly preserving 24-h urine protein specimens at room temperature without using preservatives is a safe, simple, and reliable method. This approach simplifies the pre-analytical process, eliminates potential health risks and technical issues associated with xylene use, and is strongly recommended for wide application in clinical practice.

## Data Availability

The raw data supporting the conclusions of this article will be made available by the authors upon reasonable request.
